# Revisiting the Suffixing Preference: Native-Language Affixation Patterns Influence Perception of Sequences

**DOI:** 10.1177/0956797620931108

**Published:** 2020-08-13

**Authors:** Alexander Martin, Jennifer Culbertson

**Affiliations:** Centre for Language Evolution, The University of Edinburgh

**Keywords:** language, word recognition, psycholinguistics, perception, cross-cultural differences, open data, open materials

## Abstract

Similarities among the world’s languages may be driven by universal features of human cognition or perception. For example, in many languages, complex words are formed by adding suffixes to the ends of simpler words, but adding prefixes is much less common: Why might this be? Previous research suggests this is due to a domain-general perceptual bias: Sequences differing at their ends are perceived as more similar to each other than sequences differing at their beginnings. However, as is typical in psycholinguistic research, the evidence comes exclusively from one population—English speakers—who have extensive experience with suffixing. Here, we provided a much stronger test of this claim by investigating perceptual-similarity judgments in speakers of Kîîtharaka, a heavily prefixing Bantu language spoken in rural Kenya. We found that Kîîtharaka speakers (*N* = 72) showed the opposite judgments to English speakers (*N* = 51), which calls into question whether a universal bias in human perception can explain the suffixing preference in the world’s languages.

The degree to which languages are shaped by the human cognitive system is a core question for linguists and psychologists. Indeed, many linguistic theories propose that languages are subject to constraints on learning (e.g., Chomsky, 1965; Croft, 2001), memory (e.g., Gibson, 2000; Hawkins, 2004), or perception (e.g., Blevins, 2004; Ohala, 1993). One of the major sources of evidence for these theories comes from similarities shared across many (unrelated) languages. For example, most languages use a common strategy for forming complex words: An affix is added at the end of a base (e.g., the stem “girl” becomes the plural “girl-s” by adding a suffix at the end). By contrast, the seemingly similar strategy of adding a prefix to the beginning of the stem (e.g., “un-happy”) is relatively rare. This tendency among the world’s languages has long been noted (Bybee, Pagliuca, & Perkins, 1990; Greenberg, 1957; Hawkins & Cutler, 1988; Hawkins & Gilligan, 1988; Sapir, 1921) and can be clearly seen in Table 1. More than half of the languages in that sample either predominantly use or have a moderate preference for suffixes (55%), whereas relatively few skew toward prefixes (16%).

**Table 1.** Counts of Languages From a Large Database in Terms of Their Preferences for Suffixing or Prefixing (Dryer, 2013)

Classification	Number of languages
Little or no inflectional morphology	141
Predominantly suffixing	406
Moderate preference for suffixing	123
Approximately equal amounts of suffixing and prefixing	147
Moderate preference for prefixing	94
Predominantly prefixing	58
Total	969

Researchers have sought to understand this so-called suffixing preference in terms of the human perceptual system. Hawkins and Cutler (1988) argued that the beginnings of words are most salient to the human speech-perception system, and this privileged position is reserved for the most important content: the stem. Indeed, if word recognition happens by a continual process of ruling out words that are inconsistent with what has been heard so far (Balling & Baayen, 2012; Brown & McNeill, 1966; Christiansen, Chater, & Culicover, 2016; Grosjean, 1980; Marslen-Wilson, 1987), then placing material that uniquely identifies a word earlier—typically the stem—means faster recognition. Additionally, distorting word beginnings disrupts word identification more than distorting word endings (e.g., Marslen-Wilson, 1975; Marslen-Wilson, Tyler, Waksler, & Older, 1994), and mistakes in pronunciation are easier to detect at the beginnings of words (e.g., Cole, 1973). If word beginnings are more salient and stems are more useful for identification, then it makes sense for languages to use suffixes rather than prefixes.

An alternative hypothesis relies on domain-general features of our perceptual system rather than speech perception in particular. Hupp, Sloutsky, and Culicover (2009) argued that the salience of beginnings holds for sequences of any kind. They showed that native English-speaking adults judge sequences of words, musical notes, and shapes as more similar when they differ at the end rather than the beginning. Table 2 shows example target and test sequences for syllable and shape stimuli in their experiments. Participants were asked to choose which test sequence was more similar to the target—the “prechanged” sequence, in which the initial element differed from the target, or the “postchanged” sequence, in which the final element differed. Participants consistently chose the postchanged sequence regardless of the stimulus type.

**Table 2.** Example Target and Test Stimuli Used in the Experiments of Hupp, Sloutsky, and Culicover (2009)

Domain	Target	Prechanged item	Postchanged item
Syllables	ta-te	be-ta-te	ta-te-be
Shapes	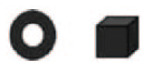	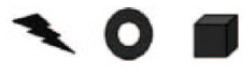	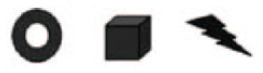

Note: In prechanged items, the initial element differed from the target; in postchanged items, the final element differed.

Hupp et al. (2009) argued that the suffixing preference found across languages thus begins with the privileged status of sequence beginnings—a domain-general feature of perception. However, their results are also compatible with an alternative interpretation, namely, that preferences are driven by prior language experience: English speakers may be attuned to differences in word beginnings because they have learned that beginnings are more informative in English or because related English words tend to differ at their ends, not because of any universal perceptual bias. This learned behavior is then transferred to other domains.

**Statement of Relevance**

Psychological scientists and linguists have long thought that languages take the forms that they do because of universal features of human cognition. For example, it has been claimed that languages tend to use suffixes (e.g., “girl-s” or “quick-ly”) more than prefixes (e.g., “un-happy”) because humans universally perceive words to be more similar when they differ at their ends than at their beginnings. However, most of the experiments testing these claims have focused on speakers of well-studied languages, with populations characterized as Western, educated, industrialized, rich, and democratic (WEIRD). Here, we tested the suffixing claim in two groups, one from a WEIRD population (English speakers on Mechanical Turk) and one from a non-WEIRD population (Kîîtharaka speakers in rural Kenya). We found that the two groups behaved very differently. This finding calls into question the claim that a universal bias in human perception can explain the suffixing preference in the world’s languages. Instead, we attribute it to historical processes.

To date, all experimental work that has tested the suffixing preference (e.g., Bruening, Brooks, Alfieri, Kempe, & Dabašinskienė, 2012; Hupp et al., 2009; St. Clair, Monaghan, & Ramscar, 2009) and most psycholinguistic work on perception, production, and retrieval of word beginnings as opposed to endings has been conducted with English speakers. If these findings reflect experience with English, then speakers of a language that is predominantly prefixing rather than suffixing may behave very differently in these tasks. Specifically, speakers of a prefixing language, in which related words tend to differ at their ends, may provide similarity judgments that are the opposite of those Hupp et al. reported for English speakers. More generally, understanding the role of human cognition in shaping language requires stepping beyond speakers of English and closely related languages. Although these languages are more familiar to many researchers and native speaker populations are easier to come by for researchers based in, for example, Europe and North America, we risk failing to see differences that can be revealed only by looking at lesser studied languages. This is reminiscent of a wider issue in psychology: the focus on populations from Western, educated, industrialized, rich, and democratic (WEIRD) societies (Henrich, Heine, & Norenzayan, 2010). Here, we looked beyond WEIRD populations, testing whether native speakers of a prefixing language—Kîîtharaka, a Bantu language spoken in rural Eastern Kenya—share the same perceptual judgments as English speakers. If they do, we will have much more convincing evidence that the suffixing preference is driven by universal features of human perception. If not, then our theories of what drives this similarity among languages may need to be revised.

## Experiment 1

### Method

#### Participants

Participants were 51 self-reported native English speakers randomly assigned to two conditions (21 in the syllables condition, 30 in the shapes condition). They were recruited through Amazon Mechanical Turk and paid 2 U.S. dollars for participating in the approximately 10-min session. This population has been documented to be more diverse in terms of age, education level, and socioeconomic background than university student populations (e.g., see Gosling, Sandy, John, & Potter, 2010). Most participants did not report knowledge of any language other than English. Crucially, no participants reported knowing a predominantly prefixing language. Informed consent was obtained prior to the testing sessions per The University of Edinburgh’s ethics protocol. We sought for our sample size to be slightly larger than those reported by Hupp et al. (2009).

#### Materials

**Fig. 1.**
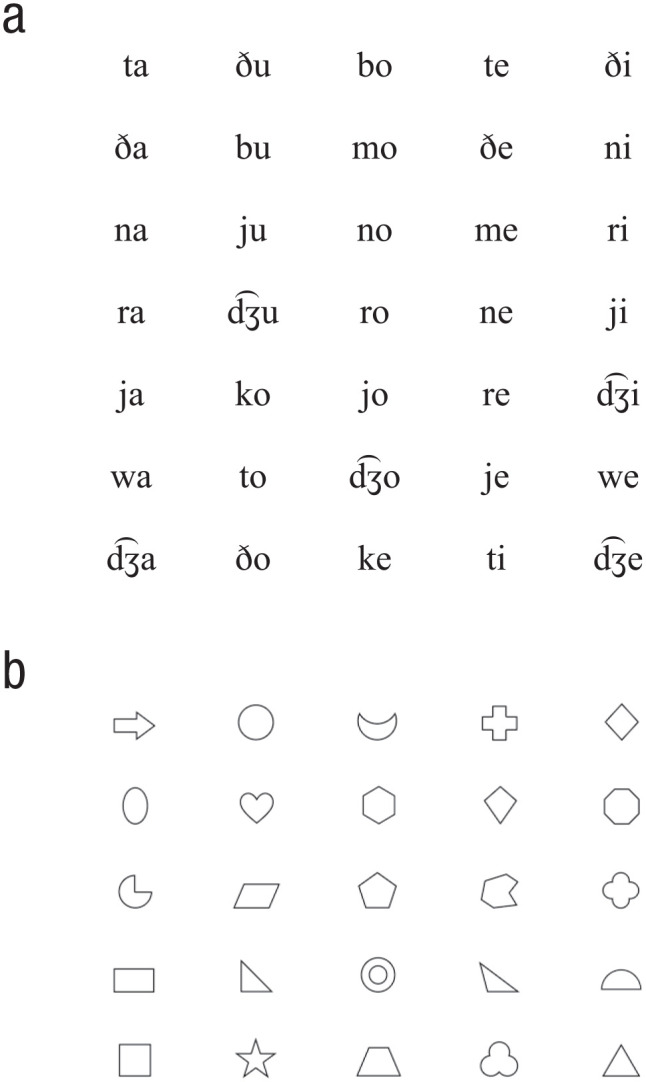
Syllable stimuli (a) and shape stimuli (b) used in the present experiments. For syllable stimuli, consonants were [t, ð, n, r, j, dƷ, w, b, k, m], and vowels were [a, u, o, e, i].

Stimuli were composed of sequences of either syllables or shapes (see Fig. 1). Sequences were either targets or test items. Target sequences always consisted of two elements (nonidentical and chosen randomly). Test items consisted of either two or three elements, depending on the type of trial, again with no elements repeating in a given sequence. There were five types of catch trials designed to ensure that participants understood the task and were paying attention. In three of the catch-trial types, participants were asked to compare sequences that were completely identical to the target with sequences that differed in (a) all elements, (b) the first element of the sequence (prechanged), or (c) the last element of the sequence (postchanged). In the remaining two types, participants were asked to compare sequences that were completely different from the target with sequences that differed in either the first element of the sequence or the last (pre- or postchanged). In critical trials, participants were always asked to compare sequences in which the first element of the sequence differed from the target (prechanged) with sequences in which the last element of the sequence differed from the target (postchanged). Note that the element that differed from the target was the same in both sequences; only its placement differed. See Table 3 for all trial types (with syllable stimuli).

**Table 3.** Example Trials of Each Type in the Present Experiments

Target	Test Item 1	Test Item 2	Trial type
tako	motako	takomo	Critical (pre- vs. postchanged)
tako	tako	jabute	Catch (identical vs. different)
tako	tako	takomo	Catch (identical vs. postchanged)
tako	tako	motako	Catch (identical vs. prechanged)
tako	takomo	jabute	Catch (postchanged vs. different)
tako	motako	jabute	Catch (prechanged vs. different)

Syllable stimuli were presented auditorily along with a picture of a loudspeaker (see the Procedure section for details). All syllables were spoken in isolation by a male speaker of U.S. English (the first author) and then concatenated (all syllables therefore received similar stress). Stimuli were designed to be used across speaker populations; therefore, the set of consonants and vowels was limited to those available for both and excluded any syllables that were class prefixes in Kîîtharaka.

#### Procedure

Participants were told they were taking part in an experiment about judging similarity. In the shapes condition, they were further instructed as follows: “You will see a sequence. Then you will see two new sequences. Your task is to decide which of the two new sequences is most similar to the original sequence. Click the one you think is right.” In the syllables condition, they were further instructed as follows: “You will hear a sound. Then you will hear two new sounds. Your task is to decide which of the two new sounds is most similar to the original sound. Click the one you think is right.” The session began with two practice trials, randomly chosen from the five catch-trial types described in the Materials section. Participants were told on these trials whether their answer was correct or incorrect. They then proceeded to complete 40 additional trials, which included 25 critical trials (with pre- and postchanged test stimuli) and 15 catch trials (three each of the five types described above).

On each trial, a target sequence appeared first. In the syllables condition, a gray loudspeaker appeared in the middle of the screen, and each syllable was played sequentially. After a 1-s delay, two grayed-out loudspeakers appeared, one on each side of the screen. After a 1-s delay, the left-hand (green) speaker was displayed in color, and each syllable of the first test sequence was played sequentially. Then after a 1-s delay, that loudspeaker was grayed out, the right-hand (blue) loudspeaker was displayed in color, and each syllable of the second test sequence was played sequentially. Participants were instructed to click on the loudspeaker corresponding to the sound they thought was most similar to the target sound (“Click the one that’s most similar to the original sound”). The side on which the loudspeaker corresponding to the correct sequence appeared was determined randomly.

In the shapes condition, both elements of the target sequence were presented together on screen. Then after a 1-s delay, the two test sequences were presented simultaneously, one on the left-hand side of the screen and one on the right. Participants were instructed to click on the sequence that they thought was most similar to the target sequence (“Click the one that’s most similar to the original”). The side on which the correct sequence appeared was determined randomly.

Note that in the original Hupp et al. (2009) experiment, the stimuli in shape sequences were displayed one at a time in quick succession. Although this makes the shape task more similar to the syllable task, it is also potentially quite difficult; participants must hold all of the shapes in memory in order to make a similarity judgment, something they do not necessarily have much experience with. We therefore chose to display the shapes in the shapes condition simultaneously.

At the end of the experiment, participants were asked whether they had any strategy during the experiment and if so to describe it. They were also asked to report other languages they knew.

### Results

#### Strategies

Most participants reported having no particular strategy (e.g., “Just paid attention to the shapes,” “I just used my intuition”). Ten participants (four in the syllables condition, six in the shapes condition) reported paying most attention to the beginnings (e.g., “I gave greater weight to the comparison samples when they contained the original sounds at the beginning of the utterance as opposed to the end,” “If the first two shapes were in the same order as the original two shapes I always picked that one as being the most similar. The other questions seemed to have objectively correct answers so I picked those answers as well”). These strategy reports function as a sanity check to make sure there was nothing unexpected about the stimuli or the task that participants used to make their judgments.

#### Catch trials

**Fig. 2.**
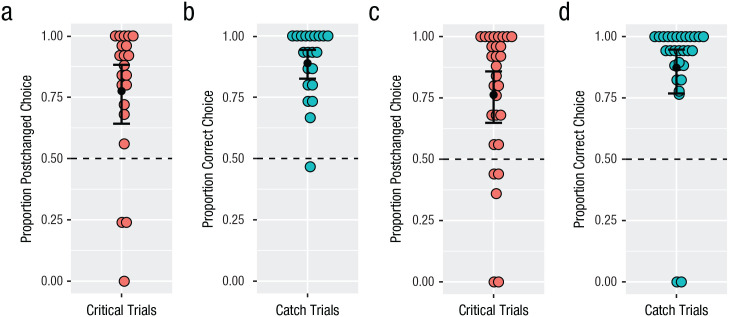
Similarity-judgment results of English speakers (Experiment 1) on critical trials (a) and catch trials (b) in the syllables condition and critical trials (c) and catch trials (d) in the shapes condition. In (a) and (c), the proportion of critical trials on which speakers chose postchanged items is shown. In (b) and (d), the proporition of correct choices in catch trials is shown. Colored dots show individual participant means, and black dots show by-participant group means. Error bars show standard errors of the mean. The dashed horizontal lines indicate chance level.

All data reported here were analyzed with mixed-effects logistic regression models run in R (Version 3.5.3; R Core Team, 2019) using the *lme4* package (Bates, 2010). All models included random by-participant and by-item intercepts (unless otherwise noted). Anonymized data and analysis scripts are available at https://osf.io/3z6kw/. Participants’ performance on critical and catch trials is shown in Figure 2. To evaluate whether participants across conditions performed similarly and above chance on catch trials, we ran two logistic mixed-effects models. The first model included an intercept term only. The second model included a fixed effect of condition. Model comparison indicated no significant improvement in fit to the data of the more complex model including condition, χ^2^(1) < 1. The significant positive intercept of the simpler model confirms that in both conditions, participants chose the correct test items at a rate that was significantly above chance (syllables: *M* = 89%, *SD* = 14%; shapes: *M* = 87%, *SD* = 25%; β = 3.10, *SE* = 0.48, *z* = 6.50, *p* < .001). This suggests that participants in both conditions understood and were able to accurately judge similarity in the task.

#### Critical trials

The critical trials were those in which the two test items were pre- and postchanged. Recall that on the basis of results reported by Hupp et al. (2009), we predicted that participants would be more likely to choose postchanged stimuli as more similar to targets than prechanged stimuli across both conditions. To evaluate this prediction, we ran two logistic mixed-effects models. The first model included an intercept term only. The second model also included a fixed effect of condition. Both models included by-participant random intercepts (including random by-item intercepts resulted in convergence issues). Model comparison indicated no significant improvement in fit to the data of the more complex model including condition, χ^2^(1) < 1. The significant positive intercept of the simpler model confirms that in both conditions, participants chose the postchanged test items more often than would be expected by chance (syllables: *M* = 78%, *SD* = 29%; shapes: *M* = 76%, *SD* = 29%; β = 2.05, *SE* = 0.37, *z* = 5.30, *p* < .001).

### Discussion

These results replicate those of Hupp et al. (2009): English-speaking participants are more likely to judge syllable and shape sequences that differ at the end as being similar to one another than sequences that differ at the beginning. Importantly, this holds despite the difference in population (lab vs. online) and stimulus presentation (sequential vs. simultaneous for shapes) between Hupp et al.’s experiment and ours.

## Experiment 2

In Kîîtharaka, as in other Bantu languages (a large language family spread across Africa), prefixal marking is abundant. For instance, in the following example, agreement prefixes on the noun, all nominal modifiers, and the verb indicate the noun class of the subject:

$$\begin{array}{l} tû\;-\;bû\mathrm{ri}\;\;\;tû\;-\;\mathrm{ra}\;\;\;tû\;-\;îrî\;\;\;tû\;-\;\mathrm{kub}î\;\;\;\mathrm{it}û\;-\;\mathrm{thi}\;-\;\mathrm{re}\\ {\mathrm{CL}}_{13}\;-\;\mathrm{goat}\;{\mathrm{CL}}_{13}\;-\;\mathrm{DISTAL}\;{\mathrm{CL}}_{13}\;-\;\mathrm{two}\;{\mathrm{CL}}_{13}\;-\;\mathrm{short}\;\;{\mathrm{AGR}}_{13}\;-\;\mathrm{leave}\;-\;\mathrm{PFV}\\ \prime \prime \mathrm{Those}\;\mathrm{two}\;\mathrm{short}\;\mathrm{goats}\;\mathrm{left}.\prime \prime \end{array}$$

The abbreviation cl stands for “noun class,” which also incorporates number. There are 17 noun classes in Kîîtharaka—nine singular and eight plural classes (as traditionally divided in the Bantu literature), referred to by numbers. agr stands for “subject agreement”; pfv stands for “perfective aspect.”

The abundance of prefixes and relatively few suffixes in Kîîtharaka (the opposite pattern of English) allows for a strong test of the hypothesis that humans universally perceive sequences that differ at their ends as more similar than those that differ at their beginnings. In addition, the Kîîtharaka-speaking population is clearly outside the WEIRD populations tested in previous work.

Kîîtharaka is spoken by approximately 175,000 people in rural Eastern Kenya (parts of Tharaka-Nithi, Embu, and Meru counties). Older Kîîtharaka speakers are monolingual, with no formal education. Younger speakers have attended school, and most can converse in Swahili (an official language of Kenya and regional lingua franca) and English. All three languages use a Latin-based, left-to-right alphabetic writing system, although knowledge and use of the three systems varies (younger speakers report texting in all three languages). The local economy is highly agrarian; most residents live in smaller remote farming communities with no electricity available outside of larger market towns. Access to the area is by a compacted dirt road some 16 km off the region’s main tarmac road (which links to the capital, Nairobi).

### Method

#### Participants

Participants were 72 native Kîîtharaka speakers randomly assigned to the syllables and shapes conditions. Most speakers were also fluent in English (which is taught to children from primary school), and nearly all were additionally able to converse in Swahili, a Bantu language with similar morphological structure to Kîîtharaka (i.e., primarily prefixing). Although the on-screen instructions were written in English, as noted above, the testing sessions were conducted in Kîîtharaka. All instructions were repeated to participants in Kîîtharaka to ensure they understood. Participants were recruited through local contacts and were paid 200 Kenyan shillings. Informed consent was obtained prior to the testing sessions per The University of Edinburgh’s ethics protocol. We sought to recruit as many Kîîtharaka speakers as possible in the time frame available and were able to test more participants than we had originally recruited for the English version of the experiment.

#### Materials

The stimuli were identical to those used in Experiment 1.

#### Procedure

The procedure used in Experiment 2 was identical to that described for Experiment 1; however, it was conducted in person (by native Kîîtharaka-speaking experimenters) using touch-screen tablets. Participants were seated in a quiet space and were instructed by an experimenter in Kîîtharaka to provide their responses by swiping the screen. For example, in a shape trial, they were instructed to “swipe the one you think is right.” At the end of the experiment, participants were asked to report whether they spoke Swahili.

### Results

#### Catch trials

**Fig. 3.**
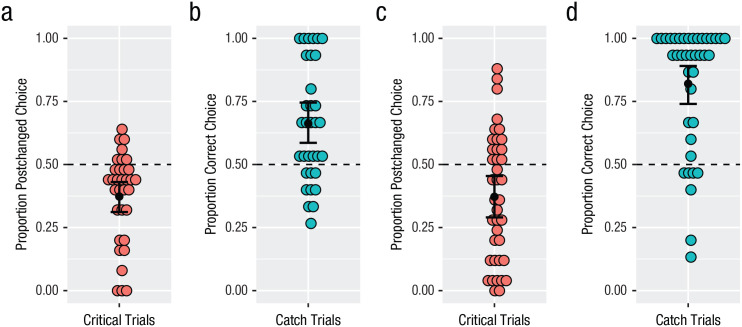
Similarity-judgment results of Kîîtharaka speakers (Experiment 2) on critical trials (a) and catch trials (b) in the syllables condition and critical trials (c) and catch trials (d) in the shapes condition. In (a) and (c), the proportion of critical trials on which speakers chose postchanged items is shown. In (b) and (d), the proporition of correct choices is shown. Colored dots show individual participant means, and black dots show by-participant group means. Error bars show standard errors of the mean. The dashed horizontal lines indicate chance level.

Participants’ performance on critical and catch trials is shown in Figure 3. To evaluate whether participants across conditions performed similarly and above chance on catch trials, we ran two logistic mixed-effects models. The first model included an intercept term only. The second model included a fixed effect of condition. Model comparison indicated a significant improvement in fit to the data of the more complex model including condition, χ^2^(1) = 8.89, *p* < .01. Although participants in both conditions performed above chance on catch trials (syllables: *M* = 66%, *SD* = 23%; β = 0.95, *SE* = 0.26, *z* = 3.64, *p* < .001; shapes: *M* = 82%, *SD* = 24%; β = 2.73, *SE* = 0.50, *z* = 5.44, *p* < .001), participants in the syllables condition performed worse. This suggests that participants in both conditions understood and were able to accurately judge similarity in the task but that the syllables task was more difficult for them. This could be because the syllables task is more memory intensive; it requires that not only the target but also the two test sequences be held in memory. Indeed, a slight numerical trend in the same direction was visible for the English-speaking participants.

#### Critical trials

To evaluate whether participants rated postchanged stimuli as more similar to targets than prechanged stimuli across both conditions, we ran two logistic mixed-effects models. The first model included an intercept term only. The second model included a fixed effect of condition. Both models included by-participant random intercepts (including random by-item intercepts resulted in convergence issues). Model comparison indicated no significant improvement in fit to the data of the more complex model including condition, χ^2^(1) < 1. The significant negative intercept of the simpler model reveals that in contrast to English speakers, participants in both conditions chose the prechanged test items more often than would be expected by chance (syllables: *M* = 37%, *SD* = 18%; shapes: *M* = 37%, *SD* = 25%; β = −0.69, *SE* = 0.15, *z* = −4.70, *p* < .001). This same effect remained true (in fact it was numerically stronger) when we restricted the analysis to participants whose performance was more 70% correct on catch trials (*n* = 41; 13 in the syllables condition, 28 in the shapes condition). There was again no effect of condition, χ^2^(1) < 1, and significantly below-chance choice of postchanged items at test (syllables: *M* = 23%, *SD* = 19%; shapes: *M* = 31%, *SD* = 27%; β = −1.30, *SE* = 0.26, *z* = −5.08, *p* < .001).

To evaluate whether English and Kîîtharaka speakers’ judgments were significantly different from one another, we ran two additional mixed-effect logistic regression models on the combined data set. One model included an intercept term only; the other included language as a fixed effect. Both models included random by-participant intercepts (including random by-item intercepts resulted in convergence issues). Model comparison confirmed that adding language significantly improved the model’s fit to the data, χ^2^(1) = 53.20, *p* < .001.

### Discussion

The results of Experiment 2 did not replicate English speakers’ preferences. Rather, Kîîtharaka speakers, whose language is heavily prefixing, showed the opposite preference: They rated prechanged sequences as more similar to targets than postchanged sequences. This held for both syllable and shape stimuli, which suggests that native-language experience affects linguistic judgments and carries over into other domains.

## General Discussion

The prevalence of languages that use suffixes (rather than prefixes) to form complex words has long been noted by linguists (e.g., Greenberg, 1957) and has led to two hypothesized explanations in which the role of human perception is central (Hawkins & Cutler, 1988; Hupp et al., 2009). Here we ran two experiments, following Hupp et al. (2009), to test the hypothesis that the beginnings of sequences are universally more salient than the ends. In our first experiment, we replicated previous findings: English speakers judged sequences of syllables *and* shapes as more similar if they differed at the end than if they differed at the beginning. However, running the same experiment with a non-WEIRD population whose native language is heavily prefixing—the Bantu language Kîîtharaka—revealed exactly the opposite pattern of results. Kîîtharaka speakers judged sequences that differed at the beginning to be more similar than those that differed at the end. These results suggest that speakers’ perception of sequences is not invariant. Rather, it is influenced by the word-formation patterns that are common in their native language. This is in line with other known cases in which linguistic experience changes perception (see Gleitman & Papafragou, 2005); for example, perception of sounds not used in our native language degrades from infancy to childhood (Werker & Tees, 1984). For both populations, preferences were similar for sequences of shapes and syllables, which suggests that native-language experience can affect perceptual judgments in other domains. Hupp et al., who used sequential presentation of both speech and shape stimuli, suggested that this similarity reflects a domain-general perceptual mechanism. However, they also acknowledged the possibility that experience with perceiving speech may transfer to other domains; indeed, in their experiments, they found that preferences for shape stimuli could be reversed through training with speech stimuli. Our case, in which shape sequences were presented simultaneously, suggests that knowledge of a left-to-right writing system (for both our populations) could facilitate this same kind of transfer (i.e., from linguistic sequences to shapes).

In principle, these findings are compatible with the idea that humans universally perceive sequence beginnings as special, but extensive experience with prefixing can override this. However, at face value, our results suggest that no universal perceptual preference for suffixes over prefixes exists. Rather, experience grouping related words on the basis of similarity either at the end or at the beginning determines how people perceive similarity in new sequential stimuli (linguistic or not). Experience with a suffixing language such as English leads to perception of beginnings as most salient for determining similarity, whereas experience with a prefixing language such as Kîîtharaka leads endings to be more salient.

In line with this, an alternative explanation of the suffixing preference across the world’s languages relies on historical processes rather than perception to drive the order of linguistic elements including affixes (Himmelmann, 2014; Hopper & Traugott, 1993; Kiparsky, 2012). Specifically, independent words commonly become affixes through phonetic reduction (e.g., the grammatical future-tense marker “’ll” from the lexical “willan,” meaning to want or to wish in the history of English; Aitchison, 2001). In many cases, affix position can be traced back to the position of an independent word before it fused with another. For example, the bias toward suffixation appears to depend on the word order of the language and the type of affix. Tense affixes (e.g., past, present, future) tend to come from independent verbs. Enrique-Arias (2002) pointed out that in languages in which verbs come last in the sentence (e.g., subject-object-verb, the most common order among the world’s languages), tense is strongly suffixing. By contrast, the suffixing preference is weaker in languages in which verbs are not last (e.g., subject-verb-object). Further, some affixes—such as subject and object agreement—do not show a suffixing preference at all, which potentially reflects their historical tendency for flexible ordering (Bybee et al., 1990; Enrique-Arias, 2002; Nichols, 1992). In addition, there may be converging mechanisms that favor fusion of following words. Himmelmann (2014) argued that prosodic breaks—for example, disfluencies such as “um” or pauses—are more likely between a grammatical word and a following lexical item (e.g., “I went to . . . um . . . school”) than between a lexical item and a following grammatical word. This could discourage fusion of would-be prefixes. In sum, the suffixing preference may in fact reflect historical accidents or independent processes concerning word order (rather than affix order).

Interestingly, there is independent evidence that prefixes offer their own cognitive or perceptual benefits. Prefixed words might be more readily recognizable—after all, prefixes occur very frequently—regardless of whether they uniquely identify a word (Marslen-Wilson et al., 1994; Pycha, 2015; Schriefers, Zwitserlood, & Roelofs, 1991). Prefixes also provide information about the kind of stem that is likely to follow (e.g., English “de-” attaches to verbal stems; Kîîtharaka “tû-” attaches to elements of the noun phrase), and learners have been shown to use the predictive power of preceding elements such as prefixes in both natural and artificial language-learning tasks (Dahan, Swingley, Tanenhaus, & Magnuson, 2000; Lew-Williams & Fernald, 2010; Ramscar, 2013; Van Heugten & Shi, 2009).

To summarize, our results challenge the notion that universal features of human perception systematically disadvantage prefixing. Although the distinct perceptual judgments of English and Kîîtharaka speakers may reflect a universal bias either augmented or diminished by language experience, there is at best no compelling evidence for that from the task we employed here and indeed, plenty of evidence to suggest that the predominance of suffixing in the world’s languages may reflect historical rather than perceptual processes. Importantly, the stark differences observed across the populations tested here suggests that explanations for the suffixing preference—or any other common feature of language—must be verified in diverse populations. More generally, when the vast majority of behavioral evidence consistent with a hypothesized universal feature of human perception or cognition comes from a single WEIRD population, then its universality should remain an open question.

## Supplemental Material

Martin_OpenPracticesDisclosure_rev – Supplemental material for Revisiting the Suffixing Preference: Native-Language Affixation Patterns Influence Perception of Sequences Click here for additional data file. Supplemental material, Martin_OpenPracticesDisclosure_rev for Revisiting the Suffixing Preference: Native-Language Affixation Patterns Influence Perception of Sequences by Alexander Martin and Jennifer Culbertson in Psychological Science

## Transparency

*Action Editor:* Rebecca Treiman

*Editor:* D. Stephen Lindsay

*Author Contributions*

J. Culbertson designed and collected the data for Experiment 1. A. Martin performed the fieldwork and trained the research assistants in Kenya to run Experiment 2. J. Culbertson analyzed the data for both experiments. Both authors wrote the manuscript jointly and approved the final version for submission.

**Open Practices:** Anonymized data, materials, and analysis scripts for the experiments are available via OSF at https://osf.io/3z6kw/. The design and analysis plans for the experiments were not preregistered. The complete Open Practices Disclosure for this article can be found at http://journals.sagepub.com/doi/suppl/10.1177/0956797620931108. This article has received the badges for Open Data and Open Materials. More information about the Open Practices badges can be found at http://www.psychologicalscience.org/publications/badges.
